# Dusk‐Dawn Asymmetries in SuperDARN Convection Maps

**DOI:** 10.1029/2022JA030906

**Published:** 2022-12-20

**Authors:** M.‐T. Walach, A. Grocott, E. G. Thomas, F. Staples

**Affiliations:** ^1^ Lancaster University Lancaster UK; ^2^ Thayer School of Engineering Dartmouth College Hanover NH USA; ^3^ Department of Atmospheric and Oceanic Sciences University of California Los Angeles CA USA

## Abstract

The Super Dual Auroral Radar Network (SuperDARN) is a collection of radars built to study ionospheric convection. We use a 7‐year archive of SuperDARN convection maps, processed in 3 different ways, to build a statistical understanding of dusk‐dawn asymmetries in the convection patterns. We find that the data set processing alone can introduce a bias which manifests itself in dusk‐dawn asymmetries. We find that the solar wind clock angle affects the balance in the strength of the convection cells. We further find that the location of the positive potential foci is most likely observed at latitudes of 78° for long periods (>300 min) of southward interplanetary magnetic field (IMF), as opposed to 74° for short periods (<20 min) of steady IMF. For long steady dawnward IMF the median is also at 78°. For long steady periods of duskward IMF, the positive potential foci tends to be at lower latitudes than the negative potential and vice versa during dawnward IMF. For long periods of steady Northward IMF, the positive and negative cells can swap sides in the convection pattern. We find that they move from ∼0–9 MLT to 15 MLT or ∼15–23 MLT to 10 MLT, which reduces asymmetry in the average convection cell locations for Northward IMF. We also investigate the width of the region in which the convection returns to the dayside, the return flow width. Asymmetries in this are not obvious, until we select by solar wind conditions, when the return flow region is widest for the negative convection cell during Southward IMF.

## Key Points

- We study dusk‐dawn asymmetries in 7 years of Super Dual Auroral Radar Network convection maps which are introduced by solar wind orientations, or data processing
- Asymmetries due to interplanetary magnetic field *B*_y_ can occur in the strength and location of the convection cells, and the return flow width
- Asymmetries due to the background model are likely to occur in the locations of the convection cells

## 1 Introduction

### 1.1 Ionospheric Convection

Ionospheric convection results from the flow of magnetic flux in the magnetosphere. The convection informs on the state of the magnetosphere and accurate measurements of convective electric fields in the ionosphere are important to correctly interpret global magnetospheric dynamics. A common way to remote sense the convection on a global scale, is to use convection maps. Convection maps are large scale maps, showing ionospheric convection around the magnetic poles. Ionospheric convection maps usually show a two‐cell convection pattern with the ionospheric plasma flowing from the dayside across the polar region toward the nightside (e.g., Greenwald et al., 1995). From there, the ionospheric plasma moves back to the dayside at lower latitudes. This convection pattern is understood to change according to the solar wind driving of the magnetosphere‐ionosphere system and nightside responses (e.g., S. Cowley, 1981a; S. W. Cowley, 1981b; S. W. H. Cowley, 1982; S. W. H. Cowley, 2000; S. W. H. Cowley & Lockwood, 1992, 1996; S. W. H. Cowley et al., 1991; M. P. Freeman, 2003; M. Freeman et al., 1991; Grocott et al., 2002, 2003, 2008; Lockwood & Morley, 2004; Milan et al., 2017; Walach et al., 2017).

Solar wind coupling of the magnetosphere‐ionosphere system not only drives activity but also asymmetries. A non‐zero interplanetary magnetic field (IMF) *B*_*y*_ component will impose a torque on the magnetic field flux tubes and affect their transport from the dayside to the nightside (S. W. Cowley, 1981b). This imposes a twist in the open magnetic flux and results in a skewed ionospheric convection pattern (e.g., Haaland et al., 2007; Ruohoniemi & Greenwald, 2005). For example, the dawn convection cell is typically smaller than the dusk cell and a positive IMF *B*_*y*_ component rotates the convection cell patterns, such that the main flow channel goes across the polar cap, from 10:00 to 21:00 MLT (e.g., Walsh et al., 2014).

Even without an IMF *B*_*y*_ component however, the convection cells are rarely symmetric about the noon‐midnight meridian. Whilst much of the ionospheric convection dynamics are attributed to solar wind driving of the magnetosphere, this lack of symmetry about the noon‐midnight meridian can be attributed to nonuniformities in ionospheric conductivity (Atkinson & Hutchison, 1978). The strong conductivity gradients in the ionosphere across the day‐night terminator squeezes the plasma flow more strongly toward the dawnside of the polar cap, which can be modeled by simulations (Tanaka, 2001). The result is a slight clockwise rotation to the convection pattern, which then results in the open flux being diverted toward the duskside of the magnetotail. The reconnection in the plasma sheet is thus also asymmetric and further introduces asymmetries into the magnetosphere (Smith, 2012). A prevailing IMF *B*_*y*_ component can introduce asymmetries which not only dictate substorm onset location but also enhance the asymmetries further (Grocott et al., 2017). Another resulting plasma flow due to asymmetries is the Sub‐Auroral Polarization Stream (SAPS), which are separate and equatorward of the convection pattern (e.g., Foster & Vo, 2002; Yeh et al., 1991). Whilst SAPS coincide with fast flows in the ionosphere, they are said to be a separate phenomenon from convection but questions around their generation mechanism remain: For example, Sangha et al. (2020) observed SAPS as a direct result of a bifurcation in the Region‐2 currents, which means they may be, at least initially, directly connected to the convection cells and thus contribute to asymmetries in the convection pattern or arise from such.

### 1.2 SuperDARN Convection Maps

Convection maps provide a useful tool in studying ionospheric convection. A well‐established way to construct these is to combine data from the Super Dual Auroral Radar Network (SuperDARN). This consists of high‐frequency coherent scatter radars built to study ionospheric convection by means of Doppler‐shifted pulse sequences and has been widely used in space physics and ionospheric research (e.g., Chisham et al., 2007; Greenwald et al., 1995; Nishitani et al., 2019; Ruohoniemi & Greenwald, 1996). SuperDARN data are continuously available from 1993, with the network having expanded over time from one radar (built in 1983) to 23 radars in the Northern hemisphere, 13 in the Southern hemisphere and more under construction. This expansion has allowed for a greater area to be covered by SuperDARN (i.e., down to magnetic latitudes of 40°) with at least 16 different look directions for each radar along which different ranges can be sampled. Line‐of‐sight measurements by this large‐scale network of radars can be combined and used to construct a picture of high‐latitude ionospheric convection on time scales of 1–2 min (Ruohoniemi & Baker, 1998). The radars can be grouped into high‐latitude radars (the original network), polar‐latitude radars (or PolarDARN), and mid‐latitude radars (or StormDARN). Nishitani et al. (2019) provides a summary from a historical northern hemisphere perspective: high‐latitude radars, at magnetic latitudes of 50°–70° were first built, starting in 1983 with the Goose Bay radar, followed by the PolarDARN radars (covering 70°–90° magnetic latitude), and the expansion to mid‐latitudes (∼40°–50°), starting in 2005 with the Wallops Island radar. Over time new radars have added to the global ionospheric convection mapping increasing the number of measurements and look directions. The SuperDARN data product most commonly used by the space science and ionospheric research community is the convection map.

In order to produce SuperDARN convection maps, five key data processing steps have to be undertaken: (a) Data from different radars are median filtered and combined onto an equal area polar grid. This allows for (b) the exclusion of data from particular radars or the specification of a range limit for the scatter. For example, slow moving E‐region scatter can and should be removed by setting the minimum range gate limit to 800 km (an empirical suggestion from Forsythe and Makarevich (2017); Thomas and Shepherd (2018)). It has become apparent that far range data beyond 2,000 km may also be problematic owing to geolocation uncertainties in the range finding algorithm (Chisham et al., 2008; Thomas & Shepherd, 2022). (c) Once the data have been filtered and combined, the latitude of the equatorward extent of the convection, or equivalently the latitude of zero electrostatic potential, is determined. This is done by fitting the data to a Heppner‐Maynard Boundary (HMB) (Heppner & Maynard, 1987; Shepherd & Ruohoniemi, 2000). (d) Data from an empirical statistical model, hereafter referred to as the “background model,” is then added to the grid. The model is parameterized by a mix of IMF conditions and solar wind velocity depending on the model. Inclusion of this data is necessary to ensure a sufficient spatial distribution of data for the subsequent step. (e) A fitting algorithm is applied which fits an electrostatic potential in terms of spherical harmonic functions to the data (Ruohoniemi & Baker, 1998; Ruohoniemi & Greenwald, 1996). To find the optimal solution for the spherical harmonic coefficients, a singular value decomposition (e.g., Press et al., 2007) is minimized. This method is also known as the “Map Potential” technique. With the expansion of the radar network, as well as data processing software improvements, the resulting data product has undergone several changes.

Grocott et al. (2012) studied the dependence of the convection patterns on the IMF using the spherical harmonic coefficients from the convection maps and found IMF *B*_*Y*_‐dependencies on the magnitude of the dawn and dusk electric potentials. Grocott and Milan (2014) studied the time‐dependence of the SuperDARN convection cells by computing the mean of the spherical harmonic coefficients for different solar wind clock angles and steadiness timescales of the solar wind. They found that the steadiness of the solar wind is important for introducing asymmetries into the convection maps: if the IMF clock angle stays in one sector for longer, asymmetries introduced by the solar wind, such as the dusk‐dawn asymmetry in the size of the convection cell become more pronounced. For example, if the IMF is pointing dawnward (*B*_*Y*_−), the dusk cell tends to enhance and the convection throat rotates toward the afternoon sector, whereas when the IMF is pointing duskward (*B*_*Y*_+), the convection throat tends to rotate toward the early morning sector. An interesting finding from Grocott and Milan (2014) is that the dawn cell is, on average, always smaller than the dusk cell under all IMF conditions.

Studies looking at dusk‐dawn convection asymmetries using SuperDARN, such as the one by Grocott and Milan (2014), have often used averaging to draw conclusions, but questions remain on how persistent some of the asymmetry features are? Furthermore, the SuperDARN data availability and data processing have changed over the years and it is reasonable to assume that these may further affect measured asymmetries: Walach et al. (2022) conducted a large scale analysis of how changes to data availability and new mapping techniques has influenced derived convection maps over the history of SuperDARN operations. The authors found that the expansion of the radar network and processing decisions can have a measurable impact on the resulting convection map data set. It was shown that when the number of backscatter points per map is high (*n* > 200), the fitting is more reliable, especially when a range limit is applied. Walach et al. (2022) also showed that for low *n* maps, the cross polar cap potential (CPCP) is often relying on the background model. This is particularly apparent when the RG96 (Ruohoniemi & Greenwald, 1996) model is used as the model bins are discrete, whereas more modern models such as TS18 (Thomas & Shepherd, 2018) and Cousins and Shepherd (2010) are able to interpolate between model bins and therefore avoid obvious model‐bias. The HMB (Heppner & Maynard, 1987), the low‐latitude boundary where the convection speeds approach 0 m/s, also suffers from this model‐dependent quantization. This previous study also showed that introducing PolarDARN radars tends to decrease the CPCP, the total electrostatic potential which the cells hold. Adding StormDARN radars to the network on the other hand, tends to increase the CPCP.

An aspect that was not covered by Walach et al. (2022) is the effect of the changes in the SuperDARN convection map data set on the dusk‐dawn asymmetries. Asymmetries in the electrostatic potential, as well as the location of the convection cells will affect the map morphologies and can therefore affect scientific conclusions drawn.

In this paper we probe the effects on dusk‐dawn asymmetries statistically to systematically isolate the effects of;

1. Differing IMF conditions for short and long timescales of IMF steadiness,
2. A limited data set with High‐latitude and PolarDARN data only,
3. A more complete data set with the addition of the StormDARN data,
4. Updating of the background statistical model from RG96 to TS18,
  and the asymmetries introduced by these.

Using the same data set as in Walach et al. (2022), we study the strength and location of the negative and positive potential cells, as well as the size of the return flow region. This allows us to investigate any large‐scale dusk‐dawn asymmetries in the convection map data set.

## 2 Methods

### 2.1 SuperDARN Data Processing

To provide a meaningful large scale comparison of different versions of the SuperDARN data set, we process Northern hemisphere data to create different versions of the SuperDARN convection maps for the same time period (2012–2018). To make SuperDARN convection maps we process the raw data using the Radar Software Toolkit (RST; SuperDARN Data Analysis Working Group et al., 2018), which can be broken down into the 5 steps summarized in Section 1.2 and described in detail in Walach et al. (2022). For Walach et al. (2022), we created 5 versions of the data set to compare to each other (D0–D4), but here we will only use 3 (D1, D3, and D4) as these are found to exhibit the most apparent differences in dusk‐dawn asymmetries. For detailed information on the data processing, we refer the reader to the appendix in Walach et al. (2022). The D1 data set includes the high‐latitude radars only with a range limit and the RG96 background model. The basic data processing is the same for all the data sets, except for the following differences (see also Table 1 in Walach et al. (2022)):

- D1: High‐latitude radars only with range limit and RG96
- D3: High‐latitude, PolarDARN and StormDARN radars (all radars) with range limit and RG96
- D4: High‐latitude, PolarDARN and StormDARN radars (all radars) with range limit and TS18

Convection maps are calculated for each data set using the varying combination of map data and background model. Data sets D1 and D3 use the Ruohoniemi and Greenwald (1996) (RG96) background model, whereas data set D4 uses the more up to date Thomas and Shepherd (2018) (TS18) background model. By including PolarDARN and StormDARN radars in data sets D3 and D4, and using the most up to date background model in D4, we simulate the historical expansion of the SuperDARN data set and updates to mapping techniques.

Range limits are added to data sets D1–D4 to attempt to reduce all possible E‐Region scatter and backscatter with higher uncertainties in projected location (Chisham et al., 2008; Forsythe & Makarevich, 2017; Thomas & Shepherd, 2018). When the range limits are applied, only backscatter data between 800 and 2,000 km is included. This is the best solution on a statistical level, and applying these range limits will remove most E‐region scatter (from ranges less than 800 km) and most of the data with higher uncertainty (from ranges greater than 2,000 km).

Comparing D1 against D4 allows us to see how the historical version of the data set compares to the most modern set‐up. This means we can clearly distinguish the asymmetries created by a limited data set with fewer radars, compared to a more complete data set with all the radars. Comparing D3 against D4 on the other hand, allows us to see the direct influence of the background model on the convection maps created with the same radar data. The RG96 model is the oldest background model available and this was built when only radar data from the Goose Bay radar was available using data from 1987 to 1993 (Ruohoniemi & Greenwald, 1996), whereas the TS18 background model was built using all the radar data from 23 radars for 2010 to 2016 (inclusive). The data used for these two background models differs not only in extent but also due to different solar wind conditions brought by the varying solar cycle. Though the sunspot number was higher for the data used for the RG96 model, the number of radars creates more differences in the model than the underlying solar cycle (Thomas & Shepherd, 2018).

### 2.2 Convection Map Parameters

Having established this archive of 2‐min resolution convection map files, we extract a set of measured parameters with which to quantify the dusk‐dawn asymmetries in the ionospheric convection maps. We extract the strength and location of the negative and positive electrostatic potential cells, as well as their latitudinal distance to the HMB, which we will from now on refer to as the return flow width. The strength of the negative and positive potentials are simply the lowest and highest potentials in the map, respectively, which is a standard output from the map potential technique. The return flow width is the latitudinal distance between the cell center (i.e., the location of the peak in the negative or positive potential) and the HMB at the same magnetic local time (MLT). The return flow region is a key indicator of geomagnetic activity. For the same potential gradient, a narrow region will mean the voltage is distributed over a smaller width leading to faster flows in the ionosphere, whereas a larger width for the same potential gradient will mean slower convective flows. An asymmetry in the return flow width between dusk and dawn, will mean that one side of the magnetosphere sees increased plasma convection in comparison to the other. Such an asymmetry will be linked to asymmetries in magnetospheric morphologies and it is thus important to characterize.

**Figure 1:**
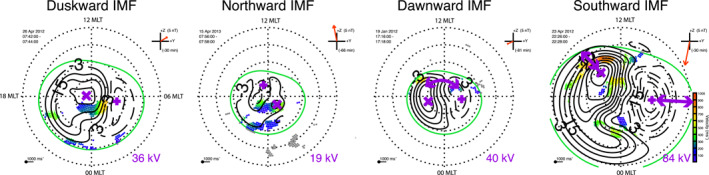
Four instantaneous convection maps showing the four solar wind conditions by which we will later sub‐sample: duskward, northward, dawnward and southward interplanetary magnetic field. Key features related to our measurements are highlighted in purple (see main text).

Figure 1 shows an example of four instantaneous convection maps, which we have chosen to illustrate the extracted measurements and the solar wind conditions by which we further sub‐sample. We have chosen example maps from time periods when the solar wind has pointed in the same solar wind direction (±15°) for more than 300 min. Each map is labeled with the relevant solar wind conditions and these are also shown by the red vector in the clock‐angle diagram to the top right of each convection map.

For each convection map in Figure 1, the magnetic pole is the center of the map, dusk is toward the left, dawn toward right, midnight toward the bottom and noon toward the top. Color‐coded vectors show the SuperDARN line‐of‐sight measurements for each map. Black solid contours show the negative potential cells, which tend to lie on the dusk‐side of the map and black dashed contours show the positive potential cells, which tend to lie on the dawn‐side of the maps. In each map, some key features related to our measurements are highlighted in purple: The duskward IMF map and consecutive maps highlight the two foci of the negative and positive convection cells as purple × and +, respectively. The contours surrounding the foci show the electrostatic potentials, which are equivalent to the convection cells. The number on the bottom right of each map, also highlighted in purple shows the CPCP. On the northward IMF map in Figure 1, we have labeled the dusk‐ and dawn sides of the maps and we see that the negative and potential cells have now switched sides across the noon‐meridian. This can be a key feature during northward, dawnward or duskward IMF. Later, we will explore the frequency at which this occurs. On the dawnward IMF convection pattern in Figure 1, we have highlighted the convection throat, where plasma flows from the dayside into the polar cap. We have not explicitly extracted this feature, but it is an important morphological constraint which we will mention again. The map for southward IMF in Figure 1 illustrates the return flow regions. The purple arrows illustrate the width of the return flow regions of the negative and the positive convection cells.

Having extracted the aforementioned parameters as a timeseries from the SuperDARN convection maps, we condense the timeseries data into probability distribution functions (PDFs) for each parameter. First, we will compare the above mentioned parameters from the negative to the positive potential cells for the D4 data set to each other. This allows us to establish a general baseline of the asymmetries present.

We then further sub‐sample the D4 data set by high *n* (*n* > 200) and times when the solar wind clock angle is purely pointing northward (0 ± 15°), dawnward (−90 ± 15°), duskward (90 ± 15°) or southward (180 ± 15°). We look at these data for when these clock angle conditions are fulfilled for a short while (*τ* < 20 min) and for a long time (*τ* > 300 min). In either case, these conditions must be fulfilled at least 90% of the time, which allows for very short solar wind deviations. This allows us to test for solar wind control of any asymmetries in the location and strength of the convection cells, as well as the importance of solar wind steadiness. Adding a limit for *n* reduces the reliability on the background model and thus allows us to isolate asymmetries that are a consequence of the solar wind conditions. We produce PDFs for these sub‐sampled data sets which allows us to readily compare the different distributions.

Using PDFs, we then compare the parameters in data sets D1 and D3 with D4, the most modern set‐up, which we use as our control data set. We compare D1 and D4 to see how the historical data set compares to the most modern set‐up. A comparison between D3 and D4 allows us to see the effects on the convection maps of changing the background model only once all radars have been added. Our approach allows us to further investigate how the expansion of the network has changed the measured parameters by comparing the figures showing D1 versus D4 to D3 versus D4.

## 3 Results

**Figure 2:**
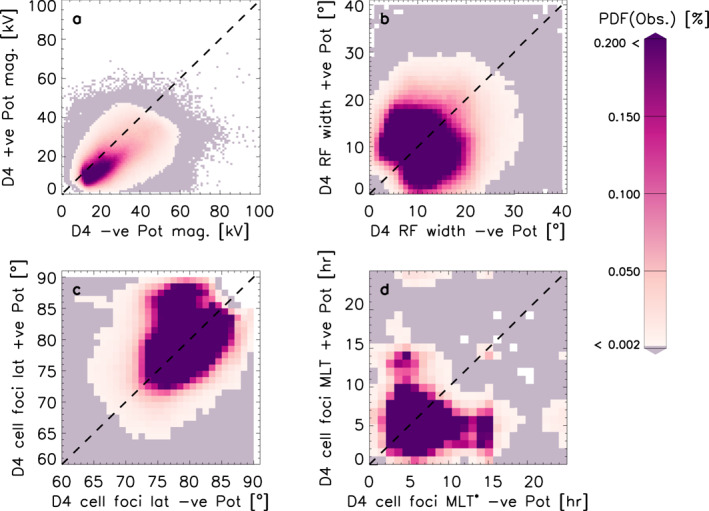
Panels (a–d) show a summary of asymmetries for D4. Panels (a–d) show the data from the negative cells against the data from the positive cells for the potential strength, the return flow width, the latitudinal location of the cell foci, and the magnetic local time location of the cell foci (MLT* = 24‐MLT), respectively.

Figure 2a–2d shows a summary of the asymmetries seen in the D4 data set, which represents the modern SuperDARN set‐up. Panel a shows the magnitudes of the negative against the positive potentials. More data lies below the line of unity (77%), as opposed to above (22%) which means the negative potential cell is more likely to be stronger. Panel b shows the return flow width of the negative and positive potential cells against each other, which show no discernible asymmetry (53% of data lie below the line of unity and 46% lie above the line of unity). Panel c show the cell foci's latitudes plotted against each other. These show some clear asymmetries. The distribution of data is skewed toward the top of the plot, which means the positive potential cell is more likely to be located near the geomagnetic pole. Overall, 47% of the data lie above the line of unity (i.e., the positive potential cell focus is closer to the geomagnetic pole), and 42% of data lie below the line of unity (i.e., the negative potential cell focus is closer to the geomagnetic pole). The remaining 11% lie on the line of unity. Panel d shows the MLT locations of the negative and positive potential cell foci plotted against each other. Here we have defined the MLT position as MLT* = 24‐MLT for the negative focus, such that the asymmetries are easily spotted. We see that the MLT location of the foci is also skewed: The negative cell focus has more data concentrated at lower MLT values (0–10 MLT* has 97% of the *x*‐axis data) than the positive cell focus at higher values (0–10 MLT has 93% of *y*‐axis data). In other words the negative cell is most likely to be located in the evening sectors on the nightside, whereas the positive cell is most likely to be located in the early morning sectors (<10 MLT). Instances where both convection foci are located on the dayside (6 < MLT < 18) only comprise 8% of all data.

### 3.1 Sub‐Sampling by Solar Wind Conditions

**Figure 3:**
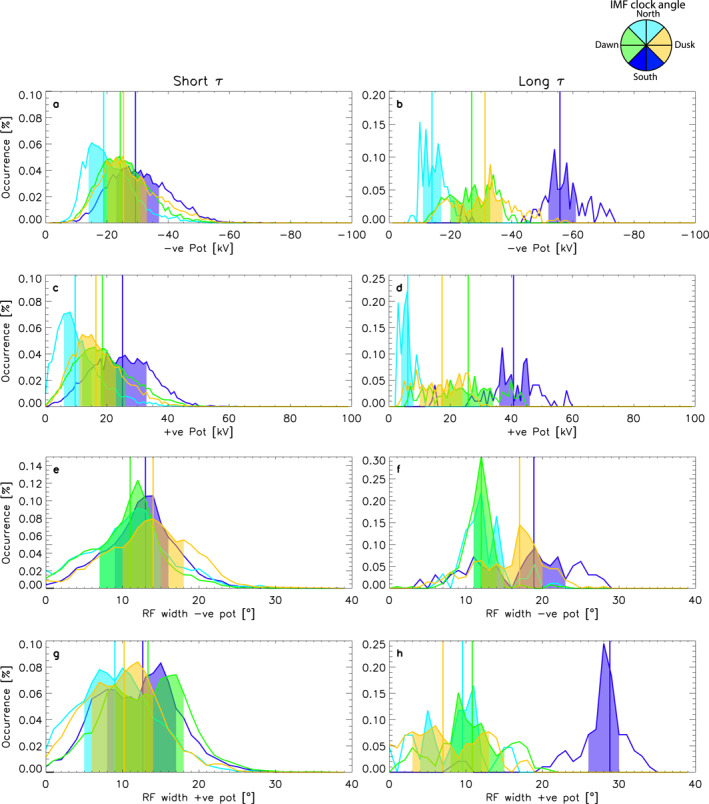
Panels (a–h) show probability distribution functions (PDFs) of D4 where *n* > 200 and the clock angle was steady for a given amount of time, the rows show different parameters (negative potential, positive potential, return flow width of the negative and positive potential cells), and each column shows the sub‐sample of the data corresponding to different steadiness timescales: up to 20 min (left) and more than 300 min (right column). The different colored PDFs correspond to varying solar wind conditions: southward interplanetary magnetic field (IMF) (−155° ≥ clock angle > 155°) in dark blue; northward IMF (−25° ≤ clock angle < 25°) in light blue; dawnward IMF (−115° ≤ clock angle > −65°) in green; duskward IMF (65° ≤ clock angle > 115°) in yellow. The colored blocks indicate the majority of the data, bounded by the lower (25%) and upper (75%) quartiles. The vertical lines indicate the medians of each distribution.

**Figure 4:**
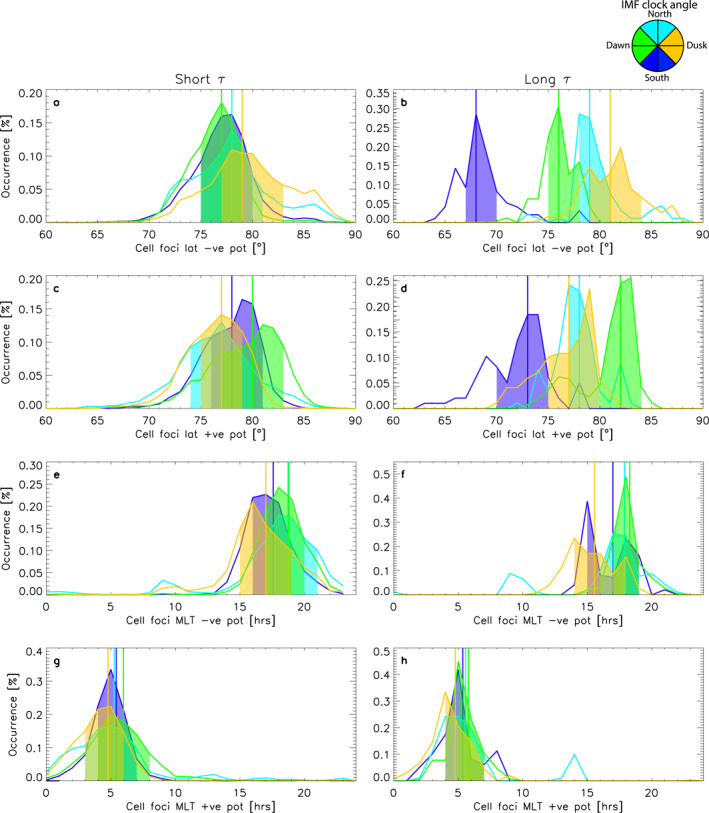
Panels (a–h) show probability distribution functions (PDFs) of D4 where *n* > 200 and the clock angle was steady for a given amount of time. The rows show different parameters which describe the cell foci locations (latitude of negative cell foci, latitude of positive cell foci, magnetic local time (MLT) of negative potential cell foci and MLT of positive potential cell foci), and each column shows the sub‐sample of the data corresponding to different steadiness timescales: up to 20 min (left) and more than 300 min (right column). The different colored PDFs correspond to varying solar wind conditions: southward interplanetary magnetic field (IMF) (−155° > clock angle > 155°) in dark blue; northward IMF (−25° ≤ clock angle < 25°) in light blue; dawnward IMF (−115° ≤ clock angle > −65°) in green; duskward IMF (65° ≤ clock angle > 115°) in yellow. The colored blocks indicate the majority of the data, bounded by the lower (25%) and upper (75%) quartiles. The vertical lines indicate the medians of each distribution.

Next, we will look at which asymmetries are controlled by solar wind conditions. For this analysis, we use a sub‐sample of the D4 data set, where *n* > 200 only, which allows us to ensure that the influence of the background model is minimized (Walach et al., 2022). This leaves us with 25% of the total data. We further split this data into times when the solar wind had a steady clock angle for up to 20 min (short *τ*) and for more than 300 min (long *τ*). We consider clock angles for southward IMF (clock angle = 180° ± 25°), northward IMF (clock angle = 0° ± 25°), dawnward IMF (clock angle = −90° ± 25°) and duskward IMF (clock angle = 90° ± 25°). Figures 3 and 4 show these data as PDFs. The left column shows short *τ* and the right column shows long *τ*. Different colors indicate the different solar wind conditions, where dark blue shows southward IMF, light blue shows northward IMF, green shows dawnward IMF and yellow shows duskward IMF. In each case, the lower (25%) and upper (75%) quartiles are highlighted by the colored blocks and the vertical lines show the medians.

Panels a–d in Figure 3 show the negative and positive potential, respectively. Panels a–d show generally that both potential cells are weakest for northward IMF and strongest for southward IMF, followed by duskward IMF for the negative potential and dawnward for the positive potential cell. The IMF *B*_*z*_ and solar wind velocity distributions for the dawnward and duskward IMF are examined further in Figure S1, which shows that they can be considered similar for dawnward and duskward IMF in each case. For long *τ* and southward IMF, we see the dark blue medians moved from −29 to −56 kV (panels a–b) and 25 to 41 kV (panels c–d). For northward IMF the distributions do not change much when the IMF timescale changes from short to long *τ*, but the differences between duskward and dawnward distributions become more pronounced for long *τ*. In all cases, the negative potentials' magnitudes are larger than the positive potentials', which means the negative potential cell holds more of the convective flow. Panels e, f, g, and h in Figure 3 show the return flow width for the negative and positive potential cells. Panel e shows that all four IMF distributions are similar for the short *τ*. All medians are between 10° and 14°, which is contrasted by the long *τ* distributions shown in panel f: Now the dark blue distribution for southward IMF has widened and the median is now highest (above 18°). The return flow width for duskward IMF is the second most likely to be wider than in panel e (above 17°), whereas the distributions for dawnward and northward IMF barely change from short *τ* to long *τ*. Panel g shows the return flow width for the positive potential cell and short *τ*. The distributions for short *τ* shown here are very similar to panel e above, except for dawnward IMF for which the median is shifted higher by a few degrees (to around 14°, as opposed to 11°). Whilst the change for dawnward IMF is fairly minimal, for southward IMF we see a more considerable change. For long *τ* (panel h), the southward IMF distribution has again shifted to the right (median at 28°), which means we are more likely to observe a wider return flow width of the positive potential cell during southward IMF.

The analysis which follows in Figure 4 is a continuation of Figure 3. Figure 4 panels a–d summarize the latitudinal location of the cell foci and panels e–h summarize the MLT location of the cell foci. Panels a and c show that the latitudinal locations of the cell foci are similar, though duskward IMF drives the negative potential cell focus much closer to the magnetic pole (panel a, yellow distribution) than any of the other distributions in panel a. In panel b, the yellow distribution is less further to the right of the plot, which means that for dawnward IMF the negative potential cell focus lies closer to the magnetic pole. The median of the yellow distribution in panel c is at 77°, whereas in panel a, it was at 79°. For long periods of duskward IMF, this pattern becomes more obvious: the negative potential cell's focus is located nearest to the pole. We see that in panel b all the other distributions have spread out too: the negative potential cell focus's latitudinal position for long periods of northward IMF has a median of 79°, for long periods of dawnward IMF the median is 76° and for southward IMF it has moved equatorward from 78° for short *τ* to 74°. In panel c, the distributions are much closer bunched together, such that they are almost indistinguishable. The distribution for the dawnward IMF conditions (in yellow) now has a median of 78° as opposed to 82° in panel a. Comparing panels c and d, the distributions stay largely the same, except for southward IMF where the cell focus moves closer to the pole as the median moves from 77° for short *τ* to 68° for long *τ*. Overall, both cell foci lie furthest away from the pole for long *τ* during southward IMF.

Panels e–h show the MLT location of the convection cell foci. Panel e shows that most of the negative potential foci lie between 15 and 21 hr, irrelevant of solar wind conditions. Panel f shows that for longer *τ* this is still the case, but we also see a secondary peak in the northward IMF and duskward IMF foci near 10 MLT. This secondary peak is also existent in panel e, but it becomes more obvious in panel f than e, as a larger proportion of the cell foci sit near 10 MLT. The positive potential cell foci's MLT location is similarly steady under different solar wind conditions: For both panels g and h, the majority of all distributions fall between 3 and 8 hr. We also see a secondary peak around 13 MLT, but only for northward IMF.

### 3.2 Sub‐Sampling by Data Set

**Figure 5:**
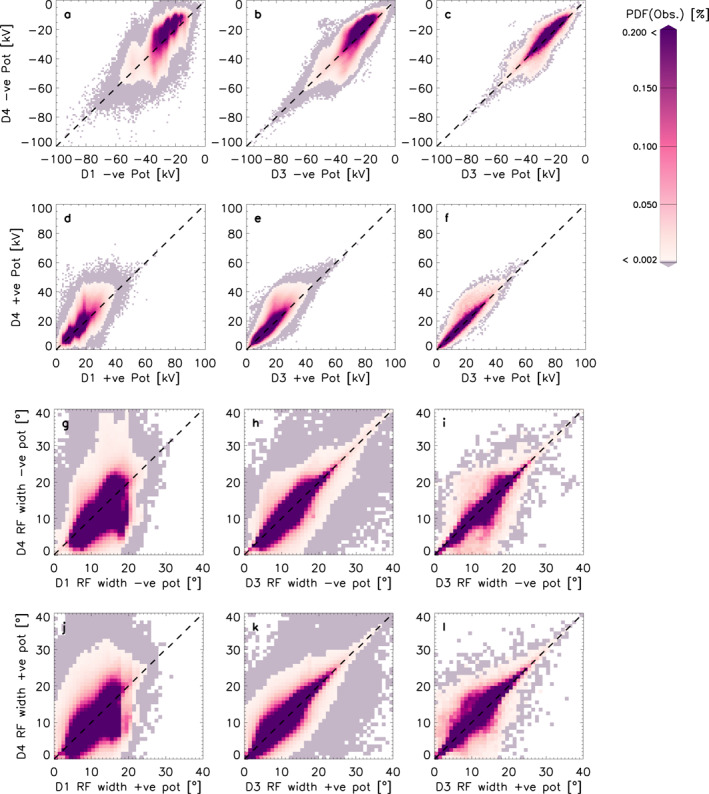
Panels (a–c) show the probability distribution functions (PDFs) of the negative potential strength for D1 and D3 against D4 and D3 (*n* > 200) against D4 (*n* > 200). Panels (d–f) show the PDFs of the positive potential strength for D1, D3 against D4 and D3 (*n* > 200) against D4 (*n* > 200). Panels (g–i) show the PDFs of the return flow width for the negative potential cell for D1, D3 against D4 and D3 (*n* > 200) against D4 (*n* > 200). Panels (j–l) show the PDFs of the return flow width for the positive potential cell for D1, D3 against D4 and D3 (*n* > 200) against D4 (*n* > 200).

Figures 5a–5c show the PDFs of the negative potential for D1 and D3 against D4 and D3 where *n* > 200 against D4 where *n* > 200 and panels d to f show the equivalent positive potential distributions. For D1 (panels a and d), the negative cell is generally stronger than the positive, which creates an asymmetry in the convection pattern. The magnitude of both potentials primarily fall within the 0–40 kV range. For panels a and d, 94% and 99% of the D1 data, respectively fall below 40 kV magnitude. When we consider which proportion of the data for D1 and D4 falls within the 0–40 kV magnitude range, this becomes a smaller portion of the data, but it is still the overwhelming majority with 85% and 98%, respectively. In panels b and e, once the entire radar network is included and we compare D3 to D4, the potential strength increases for the negative potential cell (93% of the D3 data set are now at magnitudes below 40 kV). When we introduce a backscatter echo threshold of 200 (most righthand column), we expect the convection maps to rely less on the background model and to thus be more reliable. We see this take an effect when we compare panels a, b, and d and e to panels c and f, respectively: The RG96 background model quantizes and we see vertical striations in the electrostatic potential. This is due to not enough data being available and the data processing thus relies strongly on the background model. These vertical striations were also detected by Walach et al. (2022) in the CPCP, who attributed this to the discrete binning in the RG96 model. This can also be seen to some extent in panels b and e here, though the effect is less obvious when all radars are included due to improved data coverage. When we compare panels a and d to panels c and f, the quantization effect disappears entirely. TS18 linearly interpolates between model bins, so the effect is not existent in the horizontal direction in any of panels a–f. Panels g–i show the PDFs of the return flow width for the negative potential cell and panels j–l show the equivalent for the positive potential cell. Generally, the return flow width shows little dependence on the background model but data coverage is important. Panels g and j show that the return flow width for both cells is always less than 30° for D1 in comparison to D4, which spans the full 40° range. This is due to the limited radar coverage in the D1 data set, as we observe the return flow width extending for D3 (panels h and k). Panels h and k show a reduced amount of scatter in comparison to g and j, which means the D3 return flow width is more likely to be more similar to D4's. Panels i and l have less scatter, which indicates that when data coverage is high, the return flow width becomes more stable, regardless of the background model used.

**Figure 6:**
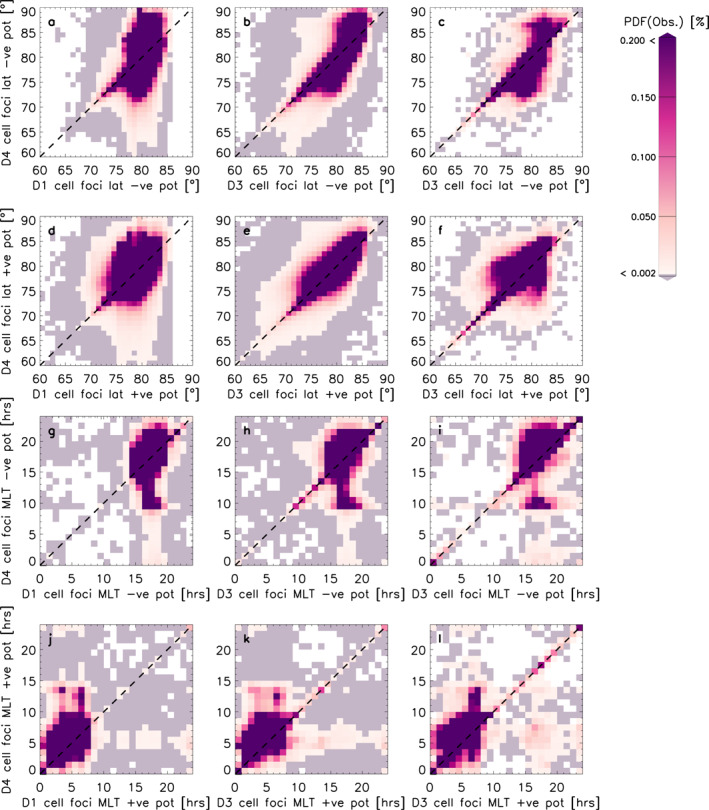
The columns are the same as in Figure 5: D3 against D4, and D3 (*n* > 200) against D4 (*n* > 200). Panels (a–c) show the probability distribution functions (PDFs) of the negative potential latitude location. Panels d to f show the PDFs of the positive potential latitude location. Panels (g–i) show the PDFs of the negative potential's magnetic local time (MLT) location and panels (j–l) show the PDFs of the positive potential's MLT location.

Figure 6 shows the PDFs for the latitudinal and MLT location of the negative and positive cell locations in the same format as Figure 5. Panels a and d show that the D4 latitudinal cell location is more variable in the D4 data set than in D1 due to the data being distributed in a fairly narrow band in the *x*‐direction in comparison to the *y*‐direction. Comparing panels a and d it seems that the positive cell is more likely to lie at lower latitudes than the negative cell as the scatter in the *x*‐direction covers a wider range in panel d. If we consider the amount of convection cell foci which lie below 75° we conclude that this the case: In panel d, 18% of the D1 convection cell foci lie below 75°, whereas in panel a this is only 3%. If we consider what percentage of cell foci in D4 and D1 lie below 75°, we find that this is 2% and 7% for the negative and positive potential cells, respectively. Panels b and e show the latitudinal location of the negative and positive potential cells for D3 against D4. In contrast to panels a and d, these show the range of the data extending to lower latitudes in the *x*‐direction. This is due to the D3 data set including all radars, which means the improved data coverage allows the cell foci to be located at a wider variety of latitudes. The percentage of negative cell foci (panel b) which lie below 75° in D3 and D4 is at 8% and for positive cell foci (panel e), this is at 12%, so the balance is similar as for panels a and d where the negative cell foci are more likely to be located at a lower latitude. Panels c and f show the subset of these data, where *n* > 200. These show a reduced version of panels b and e but no clear differences are seen between panels c and f and panels b and e, which means the asymmetries in the cell foci's latitudinal location due to the background model are existent whether or not a data threshold is introduced. There would be no background model influence if all data was distributed on or near the line of unity. Panels g–l show the negative and positive cell foci's MLT location. Panel g shows a vertical stripe between 15 and 20 MLT, where 95% of the cell foci are located in the D1 data set, whilst for D4 only 80% of data falls within this range. This tells us that there is a strong bias in the location with respect to the data set. In panel h, the vertical stripe is reduced in comparison to panel g, which means introducing more data has varied the MLT location of the negative cell foci. Now only 89% of the D3 cell foci's MLT location fall between 15 and 20 MLT. For panel i when a threshold of *n* > 200 is introduced, we see that the vertical structure reduces and instead becomes a clear secondary peak at around 10 MLT. Interestingly, we do not see a symmetric peak in the D3 foci in panel i (i.e., in the top half of the plot), which means that although we have reduced the background model's influence, this asymmetry is inherent to the background model. Panels j–l show the foci's MLT location for the positive potential cell. These show different features to panels g–i, owing to the asymmetries shown in Figure 2. In panel j, 97% of the D4 cell foci are located between 0 and 10 MLT, whereas for D1 this is almost all the data with 99%. We see again a vertical structure extending up to 15 MLT, but also a weaker horizontal extension of the main peak at 5 MLT. In panel k, the main peak becomes more defined as 98% of cell foci in D3 are contained between 0 and 10 MLT, yet both the vertical and horizontal extension of the peak remain. Panel l also shows a main peak in the cell foci's location contained between 0 and 10 MLT: 96% of the D3 cell foci with *n* > 200 are located in this range. We also see further peaks between 15 and 20 MLT but these are less pronounced and occur for both D3 and D4. This is different to the secondary peak we saw in panel i, which is primarily existent in the D4 data set. This means that sometimes the cell foci change MLT location from the main peak to the other side of the noon‐midnight meridian, but this is more likely to occur for D4 than D3, which must be due to a bias in the background model. In Figure 4 we saw that this predominantly occurs for northward and duskward IMF.

**Figure 7:**
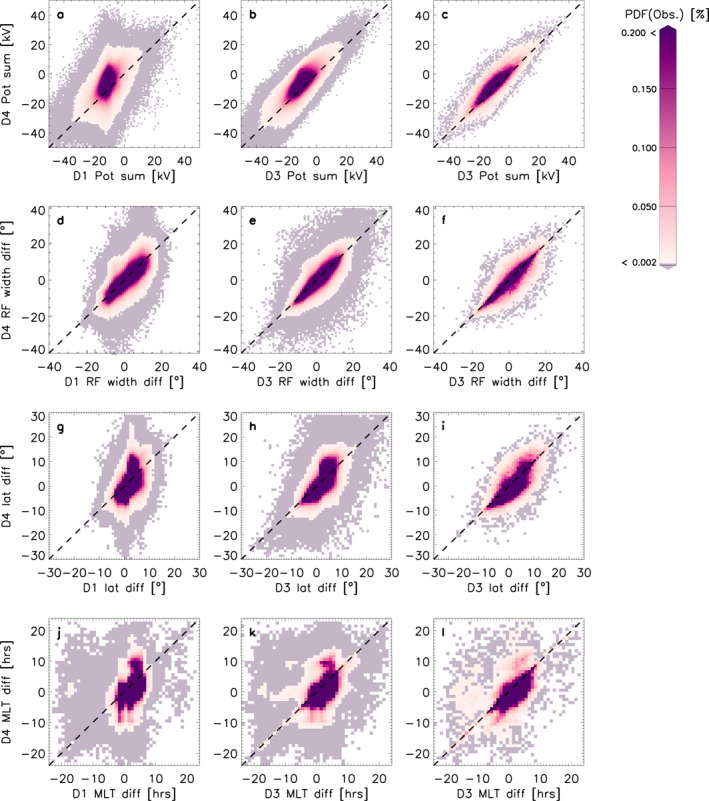
Panels (a–c) show the probability distribution functions (PDFs) of the asymmetry in the potential (the sum of the −ve potential +ve potential, for D1, D3, and D3 where *n* > 200 against D4. Panels (d–f) show the PDFs of the asymmetry in the return flow width (the difference between the −ve cell width and the +ve cell width) for D1, D3, and D3 where *n* > 200 against D4. Panels (g–i) show the PDFs of the asymmetry in the foci's latitudinal positions (the difference between the negative and positive cell foci's latitudinal positions) for D1, D3, and D3 where *n* > 200 against D4 and panels (j–l) show the PDFs of asymmetry in the foci's magnetic local time (MLT) positions (the difference between the positive cell foci's MLT* position and the negative foci's MLT position) for D1, D3, and D3 where *n* > 200 against D4.

Figure 7 shows the asymmetries in the data sets. The column layout is the same as in Figures 5 and 6 but each parameter now shows the differences between the positive and negative cells, so we can establish how the asymmetries vary. Panels a–c show the sum of the potentials (i.e., negative potential + positive potential). When this quantity is close to 0, the asymmetry between the negative and positive potentials is small. When this quantity is positive, the positive cell is dominating and when the sum is negative, the negative cell is dominating. Panel a shows that in both D1 and D4 the negative cell is mostly dominant. The large amount of scatter in panel a indicates that the asymmetries are not necessarily correlated between D1 and D4. Panel b shows the potential strength asymmetries for D3 against D4. Here, the asymmetries are largely correlated with each other. The range of the spread is within ∼20 kV from the line of unity, indicating that the background model accounts for approximately 20 kV in the variation of the asymmetry. Panel c shows the same comparison when only high *n* (>200) maps are selected. Now the scatter has reduced but overall, the PDF is similar to panel b, which means the asymmetry differences between the two background models are not fully removed.

Panels d to f show the asymmetries in the return flow width (i.e., negative cell's width ‐ positive cell's width). A negative value in these panels indicates that the positive cell's return flow region is wider than the negative cell's and vice versa. In panel d, 45% of the differences are positive for D1 and D4 and 35% are negative. This means that the negative cell's return flow width is 10% more likely to be observed to be wider than the positive cell's. This balance becomes slightly more pronounced in panel e, where 47% and 36% of the values are positive and negative, respectively. Panel f shows a reduction in scatter in comparison to panel e, but the balance between asymmetries stays approximately the same with 47% and 37% of values showing a positive and negative difference, respectively.

Panels g–i show the asymmetries in the latitudinal position of the cell foci (i.e., negative cell foci latitude—positive cell foci latitude). In panel g, most of the differences in D1 are clustered within 0 ± 10°, which means the asymmetries in the foci locations are minimal in comparison to D4. In the *y*‐direction of panel g, the asymmetries span the entire ±30° range. Panel h shows that once all radars are introduced (D3), the data spreads a wide range in the *x*‐direction also, adding to the asymmetry. In panel h we see that the asymmetries are roughly correlated with each other, but there is a large spread in values also. In panel i, where we have reduced the data set, this spread is also reduced.

Panels j–l show the asymmetries in the MLT position of the cell foci (i.e., positive cell foci MLT*—negative cell foci MLT). A positive value here means the positive cell focus is further away from the noon meridian than the negative cell focus. Panel j shows a strong asymmetry in the cell foci's MLT positions for both D1 and D4, but perhaps less in the D1 than in the D4. In panel k, we see the asymmetries are more orientated near the line of unity. In panel l, the scatter has reduced but the main data structures remain the same as in panel k: a proportion of points are clustered above the line of unity near −5 and 10 hr in D4. This means that the background model is having an effect on the asymmetries, otherwise all points would lie near to the line of unity, especially when we select by high *n* only (panels in final column).

## 4 Discussion

Our observations have uncovered a number of dusk‐dawn asymmetries in the SuperDARN convection maps. Overall, the magnitude of the negative potential cell tends to be stronger than the positive potential cell and the locations of cell foci are not symmetrically distributed. The asymmetries can largely be broken down into two groups: Asymmetries introduced by the background model and asymmetries due to solar wind control. We will now discuss the results in these contexts.

### 4.1 Asymmetries Due To Solar Wind Control

We have shown that there are clear asymmetries in the negative and positive potentials when we select by high data threshold: the negative potential is stronger, and tends to lie at lower latitudes. Since this only becomes apparent when we select maps with a high *n*, it is suggestive of a systematic asymmetry which we attribute to solar wind control of the system. This is not a new observation and there is prior evidence for this: Walach and Grocott (2019) and Walach et al. (2021) showed that during geomagnetic storms for example, when the solar wind driving is particularly strong, the convection pattern moves generally to lower latitudes, and is asymmetric with the dusk cell being stronger, which in the case of a two‐cell convection pattern is equivalent to the negative potential being stronger. Kumar et al. (2020) also showed that a strong IMF *B*_*y*_ component rotates the electrodynamical boundary between the dawn and dusk convection cells because they are linked via the field aligned current system to the ring current. They link this to alterations in the MLT distribution of ring current asymmetry, especially over timescales when the IMF *B*_*y*_ component is enhanced for ∼12 hr or more. Since data for particularly long steady IMF periods, such as the *τ* ≥ 12 hr used by Kumar et al. (2020), are binned in our study together with shorter *τ* (≥5 hr), the cell foci's MLT locations are fairly similar for dawnward and duskward IMF.

Murr and Hughes (2007) also studied IMF conditions and their effects on ionospheric convection by examining the coherence between IMF measurements from the GEOTAIL mission and ionospheric equivalent flows derived from magnetometers. Murr and Hughes (2007) found that the coherence is higher for the North‐South component and IMF *B*_*z*_ than the East‐West convection component and the IMF *B*_*y*_ component. Overall, they also found that the coherence drops by a factor of three between the periods 32 and 21 min. We therefore expect convection responses to the solar wind to be more effective for short *τ*. We find however that the PDFs differ more for long *τ* than short *τ*, indicating that the large scale features in the convection pattern are more affected by longer *τ* IMF direction. Grocott and Milan (2014) also found that the convection asymmetries become more pronounced over longer timescales, but only when the IMF *B*_*z*_ is northward. Grocott and Milan (2014) find that the negative potential cell responds similarly to solar wind forcing, but the positive potential cell does not vary much in potential for dawnward IMF. We speculate that this is due to the differing methods used by Grocott and Milan (2014), who calculated average convection patterns. This discrepancy in results is likely due to the fact that Murr and Hughes (2007) only looked at the convection throat and we have studied parametrisations of the convection pattern overall.

When we filter our data further by solar wind conditions, the convection cells are strongest during southward, followed by dawn‐ or duskward IMF, depending on the cell. Our results largely agree with those from Grocott and Milan (2014), as we find that the negative potential cell becomes on average stronger for duskward IMF than dawnward IMF and the positive potential cell becomes on average stronger for dawnward IMF than duskward IMF (see Figure 3). In this study this is more pronounced during longer intervals of steady IMF, whereas in Grocott and Milan (2014) only the negative potential cell increases strongly for long *τ* under duskward IMF. We further find that asymmetries in the location of the convection cells become particularly pronounced for northward and duskward IMF. When we filter the data for longer periods (*τ* > 300 min) of steady IMF, the location of the positive potential tends to be at latitudes of 73° for southward IMF, whereas for dawnward IMF the location tends to be nearer to 82°. For duskward IMF, the positive potential tends to be at lower latitudes than the negative potential and vice versa during dawnward IMF. These results largely match with the findings of Grocott and Milan (2014), who used SuperDARN data to calculate the average convection pattern for different clock angles and IMF timescales: Grocott and Milan (2014) also found that for duskward IMF the positive potential tends to lie at lower latitudes than the negative potential and vice versa for dawnward IMF. However, Grocott and Milan (2014) did not find that the convection pattern expands to as low latitudes as we did, but we know from Figure 6 (panels a and d) that this is due to the variation in analysis methods and to the fact that they used only data from 2000 to 2006, when no mid‐latitude radars where built in the Northern hemisphere. The results from Grocott and Milan (2014) would be closer to our D1 results, which we have not split by solar wind conditions. Our results make it clear that behind every average convection pattern, lies a multitude of possibilities. When data is averaged together, the convection maps will most likely tend to favor higher latitudes, where backscatter is more likely to be observed due to better coverage by the radar network.

We find that the return flow width differs for the negative and positive potentials, when we select by solar wind conditions: it is clearly widest for southward IMF. This is not a surprise, as we expect convection to be stronger and span a larger range of latitudes during southward IMF, especially over longer timescales of steady IMF. Walach et al. (2021) for example, showed that during the main phase of a storm in particular, when the IMF is southward, often for several hours, the return flow width becomes wider than usual. We find that the return flow width has little systematic asymmetry associated with it and we postulate that this is due to the very symmetric HMB, which is used in the SuperDARN mapping. Whilst the dayside portion of the HMB is rotated slightly clockwise toward earlier local times and is thus slightly asymmetric, but this is accounted for as the convection cell foci are on average closer to the nightside than the dayside (see Figure 2, panel d).

We find that for long periods of steady IMF, the negative and positive potentials can swap MLT sector, as they move from ∼0–9 MLT to 14 MLT or ∼15–20 MLT to 10 MLT, which means the asymmetry in how far the average foci locations are from the noon‐meridian is reduced as the swapping of MLT sectors for the positive and negative cells brings both potential locations to ±2 hr from noon. If the negative potential cell is located near dawn and the positive cell near dusk, the convection cells reverse. During long *τ*, we find that the largest asymmetry is now likely to be present under duskward IMF conditions, where the possibility of observing the potential focus location spans a large range of MLT sectors. Unfortunately, it is not possible to establish a comparison between this result and those obtained by Grocott and Milan (2014) due to their study showing an average pattern for each solar wind condition. They do however find that when the IMF has been northward for a longer period of time, a four‐cell pattern can establish, where a pair of reverse convection cells appears on the dayside at high latitudes due dual lobe reconnection, which closes open flux by reconnecting open field lines from the northern and southern hemispheres with each other (Burke et al., 1979; Greenwald et al., 1995; Imber et al., 2007; Reiff & Burch, 1985; Russell, 1972). These reverse convection cells usually appear superposed on top of the existing dual‐cell convection pattern. During intervals of northward IMF with a *B*_*y*_ component, single lobe reconnection on open field lines produces a single convection cell in the polar cap (e.g., S. Cowley, 1981a; S. W. H. Cowley et al., 1991; Jørgensen et al., 1972; Reiff & Burch, 1985; Russell, 1972; Taylor et al., 1998; Imber et al., 2007). Both dual lobe or single lobe reconnection move the peak of the negative potential cell from dusk to dawn and vice versa (e.g., Imber et al., 2007; Reiff & Burch, 1985). We are unable to distinguish between the two mechanisms here, but we do see a clear correlation with the IMF direction. Imber et al. (2007) report: “dual lobe reconnection would be expected to cease when the clock angle exceeds ±15°; at which point single lobe reconnection would be expected to recommence.” This explains why we see the negative and positive potentials swap positions not only when the IMF is purely northward, but also when it is pointing dawn‐ or duskward, though during dawn‐ or duskward IMF it occurs preferentially for short IMF steadiness intervals.

Taylor et al. (1998) used SuperDARN and Defense Metorological Satellite Programme data to show that flow reconfigurations in the ionosphere associated with northward IMF can start to occur on short timescales (∼2 min). This does however not necessarily mean a swapping of positions of the convection cell foci as these flows can be superposed on existing dual‐cell convection. Our statistics agree with the timescales shown by Taylor et al. (1998) and we show that the positional swapping of the convection cells can happen on short and long timescales of steady IMF, but is more likely to occur for longer *τ*. What is interesting is that the findings by Grocott and Milan (2014) show that the reverse convection cell only overpowers the dual convection cell after ∼240 min. This would appear in our data set as a positional swapping of the negative and positive cell foci in MLT sector, whereas we find that, statistically this can happen on shorter timescales too.

When we sub‐sample D4 for *n* > 200 and solar wind conditions, we find that the two convection cells are most likely to swap sides (i.e., the MLT of the positive potential focus is higher than the MLT of the negative potential focus) when the IMF is northward. When the IMF has been northward for a long interval (>300 min), the positional swap occurs ∼1.1% of the time, whilst these IMF (long *τ* and northward IMF) and *n* conditions are fulfilled only 0.05% overall. For the short intervals of northward IMF shown in Figure 4, this only occurs 4.1% of the time with the IMF conditions being significantly more likely to occur (IMF conditions are fulfilled 0.31% of overall data set). This means that overall, the positional swap is 23 times more likely to be observed when the IMF is pointing northward for short *τ*. For long periods of duskward IMF, the two convection cells swap MLT sectors less often: this occurs 0.37% of the time, which is reflected by the fact that these solar wind conditions are fulfilled more often (0.15% of the entire data set). Short periods of duskward IMF are statistically much more likely to occur (∼0.42% of all data) and yet, the convection cells are still not as likely to swap sides for these conditions as during northward IMF (0.95% of observable times).

This raises the question of how important the timescale of steady IMF is for the development of the reverse convection cell. In the past, different timescales have been reported for this. Imber et al. (2007) for example, observed the IMF clock angle passing gradually from −180° to 0° to 180° over the course of 3 hr, but they report that the clock angle has to be ±15° of northward IMF for dual lobe reconnection to occur. Similarly, Imber et al. (2006) estimated that the clock angle has to be ±10° for dual lobe reconnection to occur, but Imber et al. (2007) shows that lobe reconnection can occur as soon as the IMF clock angle is pointing ±15°. Here we have shown that the convection cells can swap sides on short and long timescales, but it preferentially occurs when the IMF has been northward for short periods of time due to the higher possibility of the IMF conditions being fulfilled.

### 4.2 Asymmetries Due To the Background Model

Similar to the CPCP investigated by Walach et al. (2022), we see striations in the strength of the potential cells (mainly in D1 and less obviously in D3) for the maps created using the RG96 background model. These disappear when we change the background model to TS18 (D4) or only use maps with a high data threshold (*n* > 200). As already discussed in Walach et al. (2022) this is due to the RG96 model choosing discrete bins, which the fitting algorithm will rely on when little data is available.

We find that the MLT locations of the negative and positive potentials are not evenly distributed. That is to say, they are not mirrored around the noon meridian and do not cover an equal range of MLT values. Some of this will be due to innate asymmetries in the magnetosphere, as well as solar wind control, as discussed in the previous subsection (see also Walsh et al., 2014), but there is also an asymmetry due to the chosen background model. In particular, the negative potential's focus tends to be more confined to specific MLTs in D1 and D3, but can cover a large range of MLTs in D4, which manifests itself as larger asymmetries for D4 than D3 and D1. This means the RG96 model restricts the negative potential cell to a smaller range of MLTs than TS18. This is likely due to the fact that RG96 was developed with data from only one radar, whereas TS18 used 23 geographically distributed radars. In the convection pattern, this is likely to manifest itself as a fairly stable dusk cell with a more mobile dawn cell. We find that the convection cells swap sides (i.e., lobe‐reconnection cells have established themselves) 0.6% of the time for D3 and 0.5% of the time for D4, irrespective of solar wind conditions. When we sub‐sample D3 and D4 by *n* > 200, the convection cells swap sides 1.6% of the time for D3 and 1.4% of the time for D4. As the reverse cells only occur under specific solar wind conditions, we conclude that the bias in the convection cell placement manifests itself little for times when the convection cells are strongly dependent on the IMF. It is worth noting that whilst the background model can introduce a bias, it is generally less likely to do so when a large number of datapoints is available for the fitting. Although, indicating that whilst the background model can introduce a bias, it is generally less likely to do so when a large number of datapoints is available for the fitting. This is shown in the location in MLT of the convection cell foci which takes on a more discrete peak in the PDFs (Figure 6). Figure 7 showed that this is due to a reduction in scatter and asymmetries which are brought about by the background model remain.

We further saw in Figure 7 that the asymmetries in the electrostatic potential are correlated with each other for D3 and D4 (for *n* > 200), indicating that these are driven by the data. Asymmetries in the positional placement of the foci however, remain when *n* > 200 is introduced, and they are not necessarily correlated for D3 and D4, which means there is an inherent bias in the background model.

In the average maps characterized by solar wind conditions shown by Grocott and Milan (2014), the IMF control shows that even when the IMF clock angle is pointing duskward for a prolonged time, the dusk cell's potential is always higher than the dawn cell's. Whilst we find that the negative (dusk) cell tends to hold a higher potential on average, we find that it is possible for the dawn cell to hold a higher potential than the dusk cell. Interrogating our data set, we find that for the data set using the TS18 background model (D4), the positive potential is stronger than the negative potential ∼23% of the time, whereas in D3 (which uses the RG96 background model), this only occurs in ∼10% of the convection maps. This shows that there can be considerable asymmetries introduced by the background model and depending which one is chosen, dusk‐dawn asymmetries appear to varying degrees.

## 5 Summary

In this paper we have shown that there are systemic dusk‐dawn asymmetries seen in SuperDARN convection maps. We have shown that these are due to a mixture of solar wind control of the magnetosphere‐ionosphere system and biases in the SuperDARN background models.

Observations in the data due to asymmetries introduced through solar wind control:

- When the data is filtered by solar wind conditions, the convection potentials are strongest during southward and dusk‐ or dawnward IMF. The positive potential cell is strongest during sustained periods of steady dawnward IMF, and the negative potential cell is strongest for sustained periods of steady duskward IMF. Asymmetries in the location of the potential foci become particularly pronounced for dawnward and duskward IMF.
- The negative and positive potential foci can swap positions for north‐, dusk‐ and dawnward IMF and both short and long periods of steady IMF, but it is most likely to be observed when the IMF is northward for short periods of time.
- When the data is filtered for long periods (at least 300 min) of steady IMF, the location of the positive potential can be at latitudes down to 60° for southward IMF, whereas for dawnward IMF the location is contained to above 70°. For duskward IMF, the positive potential tends to be at lower latitudes than the negative potential and vice versa during dawnward IMF.
- For long periods of steady IMF, when the reverse cells establish themselves, they move from ∼0–9 MLT to 15 MLT or ∼15–23 MLT to 10 MLT, which means their position with respect to 12 MLT reduces in asymmetry. The largest asymmetry is now likely to be present under duskward IMF conditions, where we still see a large spread away from the line of unity.
- The return flow width is similar for both the negative and positive potentials, until we select by solar wind conditions, when the return flow region is clearly widest for the negative potential under southward IMF.

Observations of asymmetries in the data due to background model:

- Clear asymmetries in negative versus positive potential when we select by a data threshold (*n* > 200): the negative potential is stronger, and tends to lie at lower latitudes.
- Striations in the strength of the potentials (primarily in the maps using the RG96 background model) due to discrete binning of the background model
- By comparing different background models and a data threshold (*n* > 200), we found the background model used biased map potential fittings by influencing the return flow width, the location of the foci and strength of convection cell potentials.
- We found that introducing a data threshold does not eliminate the bias in the fitting which introduces asymmetries in the foci locations.

Whilst we have shown general statistical results here, these uncovered asymmetries may affect the conclusions drawn in statistical studies or individual case studies. In particular, we have shown that the SuperDARN background model affects the asymmetry of the convection maps and this can to some extent be mitigated by sub‐sampling the data set by using a minimal scatter‐echo threshold. However, using a threshold does however not eliminate all asymmetries: The positional placement of the cell foci in particular exhibits asymmetries that are bias due to the background model. This result means that asymmetries presented in older SuperDARN studies (using the RG96 background model) could have been influenced by the background model.

## Supporting information

Figure S1

## Data Availability

All data used for this study are available open source. The authors acknowledge the use of SuperDARN data. SuperDARN is a collection of radars funded by national scientific funding agencies of Australia, Canada, China, France, Italy, Japan, Norway, South Africa, United Kingdom, and United States of America, and we thank the international PI team for providing the data. The authors acknowledge access to the SuperDARN database via the British Antarctic Survey (https://www.bas.ac.uk/project/superdarn/#data). Other data mirrors are hosted by the Virginia Tech SuperDARN group (http://vt.superdarn.org/) and the University of Saskatchewan (https://superdarn.ca/data-download). The Radar Software Toolkit (RST) to process the SuperDARN data can be downloaded from Zenodo (https://doi.org/10.5281/zenodo.1403226 and references). All solar wind data used to process the data were downloaded from NASA's SPDF Coordinated Data Analysis Web (https://cdaweb.gsfc.nasa.gov/index.html/). The processed data used for this publication is available under the https://doi.org/10.17635/lancaster/researchdata/571 (Walach, 2022).
