# Supplementary material for: Dusk‐Dawn Asymmetries in SuperDARN Convection Maps
Source: J Geophys Res Space Phys. 2022 Dec 20;127(12):e2022JA030906. doi: 10.1029/2022JA030906 (PMC10078218; doi:10.1029/2022JA030906)
Supplement: Supplementary file 1 — Figure S1 [file JGRA-127-0-s001.pdf]

# Supporting Information for "Dusk-Dawn Asymmetries in SuperDARN Convection Maps"

M.-T. Walach[1], A. Grocott[1], E. G. Thomas[2],F. Staples[3]

[1]Lancaster University, Lancaster, LA1 4YW, UK

[2]Thayer School of Engineering, Dartmouth College, Hanover, NH, USA

[3]Department of Atmospheric and Oceanic Sciences, University of California, Los Angeles, CA, USA

**Contents of this file**

1. Figure S1

**Introduction** This document contains one Figure to support the information in the main manuscript.

**Figure S1.** Figure S1 shows the IMF $B_z$ and solar wind speed distributions for the subsampled data for duskward (yellow) and dawnward (green) IMF. These are chosen in the same way as in the main mauscript. As in the manuscript, the left column shows the distributions for short $\tau$ and the right column shows the distributions for long $\tau$. The top row shows IMF $B_z$ and the solar wind speed is shown in the bottom row. The distributions for both IMF $B_z$ and the solar wind speed can be considered to be functionally the same in each case of duskward and dawnward IMF. The differences between short and long $\tau$

---

Corresponding author: M.-T.. Walach, Lancaster University, Lancaster, LA1 4YW, UK (m.walach@lancaster.ac.uk)

are primarily in the occurrence, such that long $\tau$ has fewer datapoints but they can be considered to be the same for the purpose of this study. The medians for long $\tau$ are slightly below 0 nT for the IMF $B_z$and slightly lower for the solar wind speed in comparison to short $\tau$.

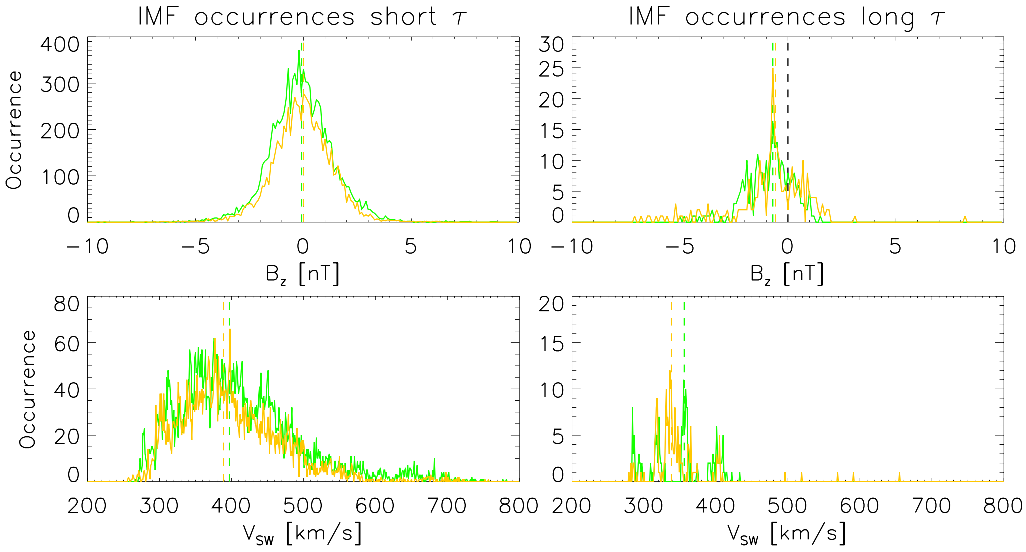


**Figure S1.** IMF occurrence distributions (green = Dawnward IMF (i.e. negative $B_y$) and yellow = Duskward IMF (i.e. positive $B_y$), as defined in the paper) for IMF $B_z$ (top row) and the solar wind speed (bottom row) for short periods of solar wind steadiness (left column) and long periods of solar wind steadiness (right column). Dashed lines show the medians.
